# Intimomedial mucoid degeneration causing aortic and renal artery aneurysms in a young adult

**DOI:** 10.5830/CVJA-2015-079

**Published:** 2016

**Authors:** Charle Viljoen, Patryk Szymanksi, Naeem Osman, Kristin Lorenc Henning, Paul ScholtZ, Brian Rayner, Nadraj Naidoo

**Affiliations:** Department of Medicine, Groote Schuur Hospital and University of Cape Town, South Africa; Department of Medicine, Groote Schuur Hospital and University of Cape Town, South Africa; Division of Anatomical Pathology, National Health Laboratory Service, Groote Schuur Hospital and University of Cape Town, South Africa; Division of Radiology, Groote Schuur Hospital and University of Cape Town, South Africa; Division of Radiology, Groote Schuur Hospital and University of Cape Town, South Africa; Division of Nephrology and Hypertension, Groote Schuur Hospital and University of Cape Town, South Africa; Division of Vascular Surgery, Groote Schuur Hospital and University of Cape Town

**Keywords:** intimomedial mucoid degeneration, aortic aneurysm, dissection

## Abstract

Intimomedial mucoid degeneration (IMMD) is characterised by aneurysm formation following mucin deposition in the intima and media, with elastic tissue degeneration of the arterial wall. We present a case of a young adult who developed a diffusely aneurysmal aorta and its major branches, which was histopathologically confirmed as intimomedial mucoid degeneration, and a review of the literature. This case report attempts to raise the awareness of the reader to this rare cause of aortic aneurysm and to the bleeding diathesis associated with IMMD that may complicate surgery.

## Abstract

Intimomedial mucoid degeneration (IMMD) is a rare vascular disorder characterised by the deposition of mucin in the intima and media, which leads to elastic tissue degeneration and aneurysm formation of the arterial wall.^1^-^4^ Although the condition was initially thought to involve only the aorta, subsequent publications have reported IMMD to affect the major branches of the aorta, as well as smaller vessels such as the coronary and brachial arteries.^1^,^5^,^6^-^8^ The aneurysms in IMMD usually have a saccular or fusiform morphology and cause symptoms related to the location of the aneurysm.^6^,^8^

Surgery is often complicated by a bleeding diathesis distinct from disseminated intravascular coagulation (DIC), but which resolves after surgical treatment of the diseased vessel.^2^ Meticulous surgical technique is of paramount importance.^2^,^6^ Peri-operatively, the coagulation profile and platelet function should be carefully monitored and diligently corrected.

## Case report

A healthy 18-year-old male presented to the emergency unit with a two-week history of coughing with increasing dyspnoea and a feeling of ‘heaviness on the chest’. On examination, a blood pressure measurement of 177/105 mmHg was noted, with good-volume regular pulses that were equal and present throughout, with no bruits. The jugular venous pressure was not elevated and the apex was undisplaced. Heart sounds were normal, but auscultation of the chest revealed crackles in the lung bases. A pulsatile mass was palpated in the epigastrium.

**Fig. 1.**
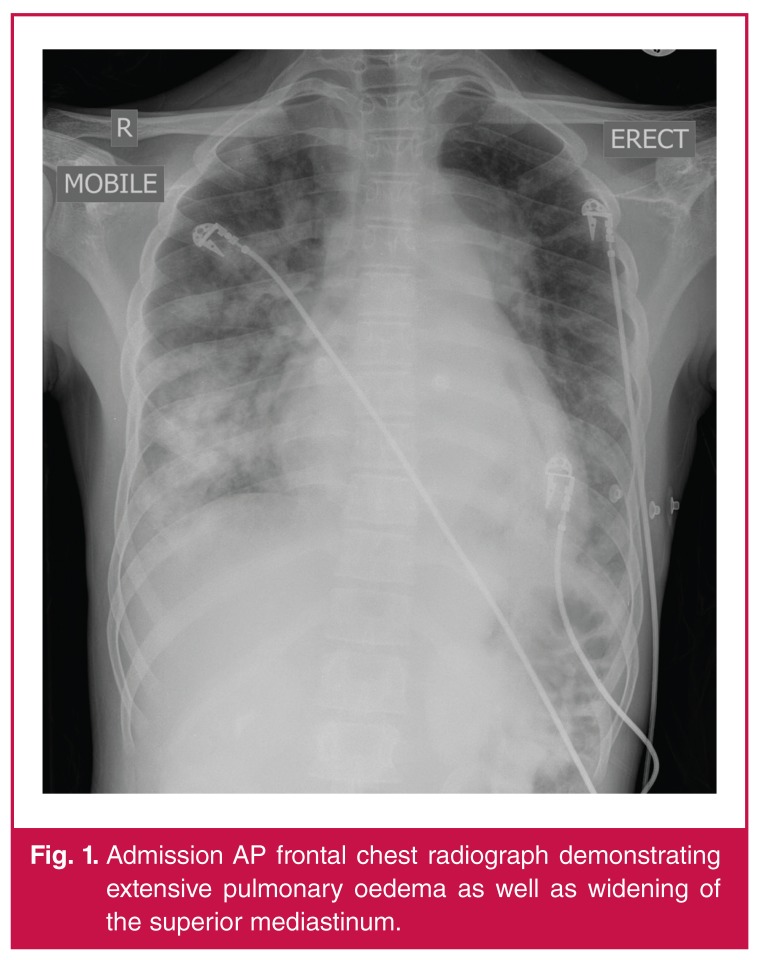
Admission AP frontal chest radiograph demonstrating extensive pulmonary oedema as well as widening of the superior mediastinum.

The admission chest radiograph revealed pulmonary oedema and a widened mediastinum (Fig. 1), with the lateral film confirming dilatation of the descending thoracic aorta. Blood work returned with Na 142 mmol/l, K 4.7 mmol/l, urea 15.7 mmol/l and creatinine 306 μmol/l. The white blood cell count was 16.82 × 109 cells/l, haemoglobin 8.7 g/dl, mean cell volume 72.4 fl and platelets 338 109 cells/l. The C-reactive protein (CRP) was 210 mg/l and erythrocyte sedimentation rate (ESR) was 111 mm/h. HIV and syphilis serology returned negative.

**Fig. 2.**
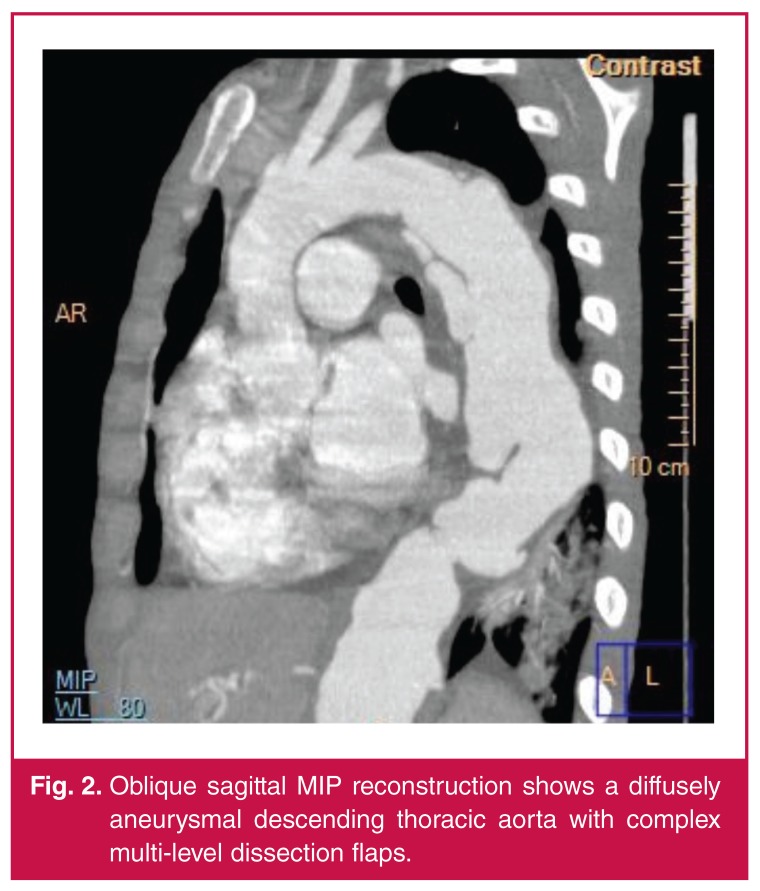
Oblique sagittal MIP reconstruction shows a diffusely aneurysmal descending thoracic aorta with complex multi-level dissection flaps.

**Fig. 3.**
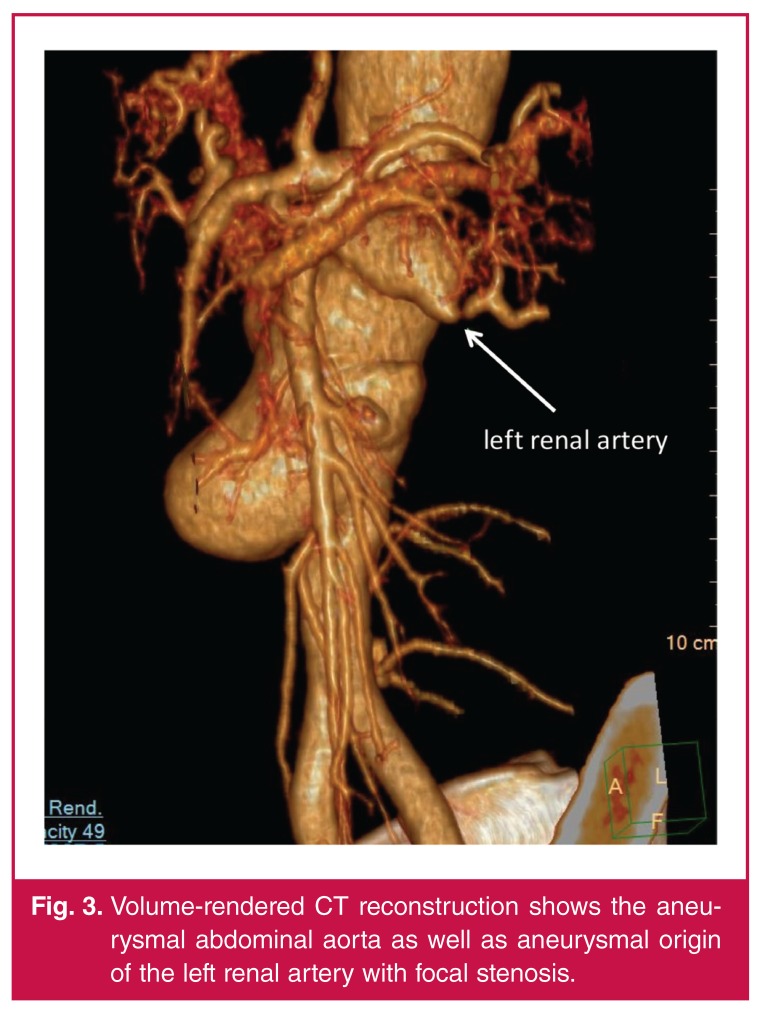
Volume-rendered CT reconstruction shows the aneurysmal abdominal aorta as well as aneurysmal origin of the left renal artery with focal stenosis.

A computerised tomographic angiogram (CTA) showed that the descending thoracic aorta was aneurysmal throughout its course (maximum diameter 46 mm), with multiple complex dissection flaps (Fig. 2). The abdominal aorta was also aneurysmal with a large lobulated aneurysm below the level of the superior mesenteric artery (maximum diameter of 52 mm). The diseased aorta was diffusely thick walled with no calcification. Both renal arteries arose from the lobulated aneurysm and the left renal artery origin was noted to be aneurysmal (Fig. 3). There was poor contrast filling within both renal arteries proximally, likely related to dissection. Further aneurysms involved the right subclavian, left common carotid and right superficial femoral arteries.

An echocardiogram showed mildly impaired left ventricular function, but normal valves. A DMSA scan indicated a differential glomerular filtration rate of 4 ml/min and 15 ml/min to the right and left kidney respectively

**Fig. 4.**
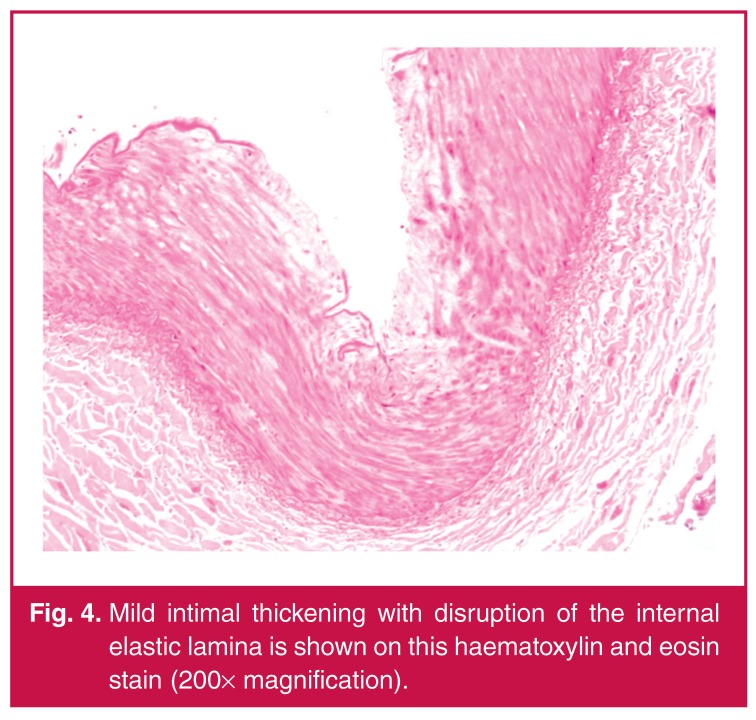
Mild intimal thickening with disruption of the internal elastic lamina is shown on this haematoxylin and eosin stain (200× magnification).

**Fig. 5.**
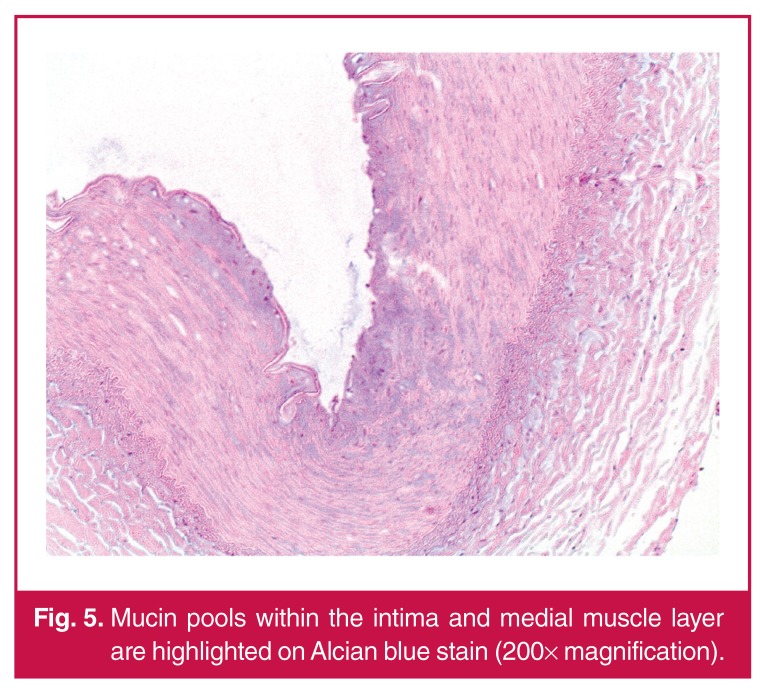
Mucin pools within the intima and medial muscle layer are highlighted on Alcian blue stain (200× magnification).

**Fig. 6.**
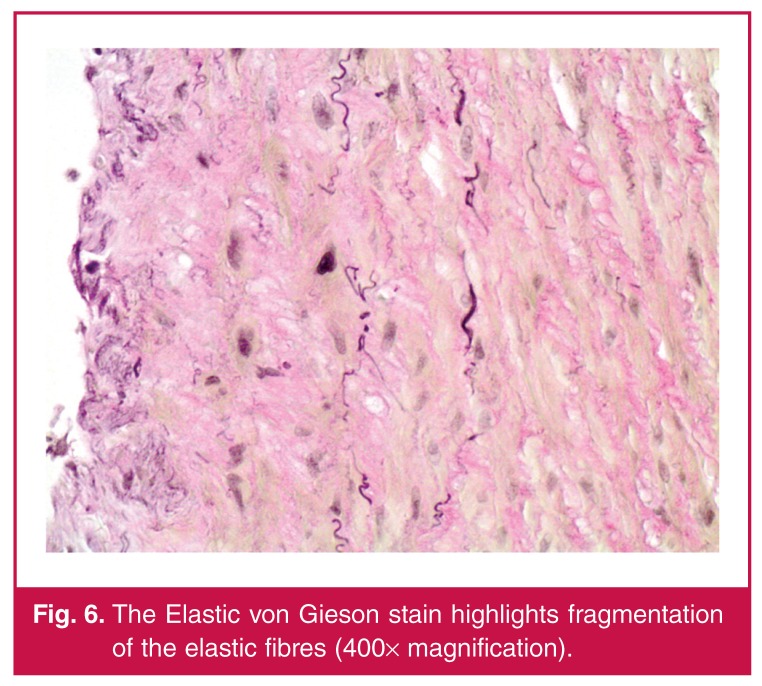
The Elastic von Gieson stain highlights fragmentation of the elastic fibres (400× magnification).

In spite of optimal blood pressure management, his renal function continued to decline. Haemodialysis was commenced prior to staged repair of a complex type 2 thoracoabdominal aneurysm. The first stage involved a left renal auto-transplantation. Histology of the left renal artery, as demonstrated in Fig. 4, Fig. 5 and Fig. 6, showed mucin accumulation within the intimal and medial layers with disruption of the elastic laminae. There was no evidence of vasculitis or atherosclerosis. The overall features were in keeping with a diagnosis of intimomedial mucoid degeneration.

The patient’s renal function and blood pressure improved postoperatively. However on the third day after admission his haemoglobin dropped and abdominal distension was noted. A repeat CTA indicated haemorrhage around the autotransplanted kidney with possible leakage of the abdominal aneurysm. The patient received an emergent hybrid repair of the extensive thoraco-abdominal aneurysm. The procedure involved debranching of the coeliac artery, superior mesenteric artery (SMA) and right renal artery, with extensive stent-graft repair using four overlapping aortic stent grafts.

Prior to the stent-grafting, the coeliac artery and SMA were revascularised with a bifurcated prosthetic graft, using the right common iliac artery as the donor vessel. Complex repair of the right renal artery followed, with subsequent right renal auto-transplantation onto the iliac vessels. Formal closure of the abdomen was performed three days later, at which point it was found that there was minimal thrombus in the abdominal aneurysm.

The patient subsequently had an uneventful course. His creatinine level improved to 73 μmol/l (MDRD estimated glomerular filtration rate was 117 ml/min/1.73 m2) and his blood pressure normalised without additional antihypertensive medication. He was discharged in good health and was attending the vascular out-patient clinic regularly, with normal renal function.

## Discussion

The condition was first described in South Africa, with the earliest cases dating back to a publication by Pepler in 1955.^9^ The term ‘intimomedial mucoid degeneration’ was first used in 1977 by Decker *et al.* from Johannesburg in their case series of nine patients with aortic aneurysms.^1^ Due to the lack of understanding of its aetiology, the condition was defined in pathological terms describing its histological features.^1^,^10^,^11^

In 1993 it became apparent that IMMD also had extraaortic manifestations, when Cooper *et al.*. from Durban published a series of six cases in which the subclavian, common carotid, mesenteric and iliac arteries were found to have IMMD.^11^ In our case the right subclavian, left common carotid, right superficial femoral and left renal arteries were involved. Recent reports have also shown IMMD to affect smaller vessels, such as the coronary, brachial, dorsalis pedis and temporal arteries.^5^,^7^

Although early publications reported IMMD to be confined to predominantly female black South Africans, subsequent publications from India and Europe demonstrated that the disease is not limited to the African population.^4^,^8^,^12^ This is illustrated in our case as our patient was of mixed ancestry and male. Various studies have shown that aneurysms in IMMD affect a younger population group than what is found in conventional non-specific degenerative aneurysms.^1^,^4^,^6^ As in this case, patients with IMMD have a high prevalence of hypertension, which aggravates the elastic tissue breakdown, resulting in aneurysm formation.^1^,^4^,^6^,^9^,^11^

Patients with IMMD present with localised symptoms related to the position of the aneurysms.^7^,^9^ Presenting symptoms include abdominal and back pain, presence of a pulsatile mass, limb claudication and symptoms related to aneurysm rupture.^6^ This could be explained by the most common sites of involvement being the infra-renal aorta, followed by the thoracic aorta, subclavian, common carotid and common iliac arteries.^6^ Our patient did not present with abdominal or back pain, however, his chest discomfort could be explained by the local effects of the aneurysmal descending thoracic aorta.

The morphological characteristics of the aneurysms found in patients with IMMD are usually of the fusiform or saccular types.^8^ Various imaging modalities, namely duplex ultrasound, CTA and/or magnetic resonance angiography, are used to determine the extent of disease, and whether or not there is an element of dissection.^6^,^8^

The principle histological features of IMMD include intimal and medial thickening resulting from accumulation of mucin pools, which in turn leads to fragmentation and aggregation of elastin fibres, as illustrated by our case.^8^,^11^ The weakened wall structure finally results in aneurysm formation.^2^,^11^ A striking feature on histological examination is the absence of any inflammatory reaction.^1^ The features are distinct from cystic medial necrosis, in which only the media is affected by the mucin accumulation.^1^,^4^ Cystic medial necrosis is also typically confined to the aorta, whereas IMMD has been found to involve extraaortic vessels.^1^,^11^ Extra-aortic disease in IMMD may also be found without any aortic involvement. 2,11

A distinctive feature of IMMD is the paucity of luminal thrombus in the aneurysm sac.^7^ Patients often suffer from bleeding intra-operatively.^8^ This bleeding diathesis is aggravated by surgical manipulation and is reversed once the aneurysm is repaired. It is therefore postulated that there is a primary fibrinolytic process that originates from the diseased aneurysm, which might explain why a thrombus is seldom found in IMMD, as was the case in our patient, and why occlusive disease is a rare finding, apart from a few reports in the literature.^4^-^6^,^8^

This fibrinolytic process in IMMD is distinct from DIC.^2^ Patients with IMMD are found to have decreased levels of platelets and fibrinogen, as well as factors V and VIII.^2^ This leads to accelerated fibrinolysis, often manifest as reduced euglobulin lysis time.^2^ In contrast to DIC, positive fibrin monomer and increased D-dimer levels are not seen in IMMD.^2^,^6^,^8^ The surgical team needs to be aware of the bleeding tendency of patients with IMMD.^6^ Also, the arterial wall often appears friable with a tendency to dissect easily during suture repair. Meticulous suture technique is therefore essential.^6^,^8^ The coagulation profile and platelet function should be carefully monitored peri-operatively and diligently corrected.^8^

A single case report by Katz *et al.* describes endovascular repair of a gluteal artery aneurysm secondary to IMMD.^2^ However, the standard of care in the treatment of IMMD-related aneurysms is open surgical repair.^6^,^8^ At our institution, we have treated a few patients with IMMD-related thoracoabdominal aneurysms with hybrid procedures, comprising visceral debranching and revascularisation followed by extensive thoraco-abdominal stent grafting.

The prognosis depends on the extent of disease and the time of presentation.^8^ Acute presentation with dissection has higher morbidity and mortality rates.8 Adverse outcomes are related to major blood loss requiring massive blood transfusions and the associated complications, as well as multi-organ failure secondary to shock.^6^

At the time of submission for publication, this case report possibly represents the first described case of IMMD affecting the renal artery.

## Conclusion

Although IMMD is a rare vascular disorder, it forms part of the differential diagnoses in aortic aneurysm, especially in the younger population and in the absence of conventional risk factors associated with non-specific degenerative aneurysms. Clinicians managing patients with IMMD should be aware of the bleeding diathesis associated with this condition.
